# Day and night nurse staffing levels and hospital-associated disability in older adults in Japan: a retrospective cohort study

**DOI:** 10.1093/ageing/afaf217

**Published:** 2025-08-06

**Authors:** Noriko Morioka, Mutsuko Moriwaki, Christina Saville, Atsushi Miyawaki, Kiyohide Fushimi, Peter Griffiths

**Affiliations:** Department of Epidemiology and Biostatistics, National Institute of Public Health, Wako, Japan; Department of Health Policy and Informatics, Institute of Science Tokyo Graduate School of Medical and Dental Sciences, Tokyo, Japan; Quality Management Center, Institute of Science Tokyo, Tokyo, Japan; Health Workforce and Systems, School of Health Sciences, University of Southampton, Southampton, UK; Public Health Research Group, Institute of Medicine, University of Tsukuba, Tsukuba, Japan; Department of Health Policy and Informatics, Institute of Science Tokyo Graduate School of Medical and Dental Sciences, Tokyo, Japan; Health Workforce and Systems, School of Health Sciences, University of Southampton, Southampton, UK

**Keywords:** hospital-associated disability, nurse staffing, older adults, nursing workforce

## Abstract

**Background:**

Hospital-associated disability (HAD) in older adults often results from preventable factors. Nurse staffing at the ward level is a key modifiable factor in mitigating HAD.

**Objective:**

To investigate the relationship between nurse staffing shortfalls during typical day and night shifts and the risk of HAD in older adults in acute care hospitals.

**Design:**

Retrospective cohort study.

**Setting:**

General medical or surgical wards in nine acute care hospitals in Japan.

**Subjects:**

Hospital admissions of patients aged ≥65 years.

**Methods:**

Electronic claims data and daily ward-level nursing rosters were used to measure nurse staffing shortfalls based on the patient-to-nurse ratio relative to the annual mean.

**Results:**

Among 57 498 hospital admissions [23 981 (41.7%) female, median age 76 (interquartile range [IQR] 71–76) years], 30 507 (70%) were functionally independent at admission. Functional deterioration occurred in 15 458 (26.9%) admissions. The median (IQR) patient-to-nurse ratio across the whole day was 6.11 (5.54–6.59), 3.70 (3.34–3.90) for the day shift, and 9.38 (8.57–10.37) for the night shift. The median (IQR) deviation from typical ward-level staffing was −0.21 (−0.64 to 0.20) for the whole day, −0.23 (−0.56 to 0.09) for the day shift, and 0.01 (−0.51 to 0.49) for the night shift. For each patient above the mean patient-to-nurse ratio, the risk of HAD increased by 7%.

**Conclusion:**

Attention to the deviations from the usual staffing levels for both day and night shifts may be important in efforts to reduce the risk of HAD in older adults.

## Key Points

- Hospital-associated disability (HAD) is common among older adults and often results from preventable factors.
- A key modifiable factor in preventing HAD is nurse staffing at the ward level, which enables nurses to deliver regular and fundamental nursing care activities to patients.
- The specific association between nurse staffing levels and HAD is unclear.
- We demonstrated that shortfalls in nurse staffing during both day and night shifts were associated with a higher risk of HAD in older adults.
- Attention to the deviations from the usual staffing levels for both day and night shifts may be important in efforts to reduce the risk of HAD in older adults.

## Introduction

Hospital-associated disability (HAD) is defined as a loss of the ability to independently perform one or more activities of daily living (ADLs) following hospitalisation [1] Globally, a meta-analysis reported that the prevalence of HAD among older adults hospitalised was approximately 30%–37% [2, 3]. HAD represents a significant problem for older adults, including an elevated risk of nursing home placement after discharge, a higher likelihood of hospital readmission [4] and long-term loss of mobility [5, 6].

HAD is caused by various factors, including individual factors such as medical illness, ADL impairments and cognitive impairment at admission, as well as hospital-associated factors such as environmental factors, reduced mobility and enforced dependences [7, 8]. To prevent HAD, interventions focused on hospital-associated factors have proven effective. This includes regularly assessing patients’ functional status and risk for HAD, providing health education to patients to promote exercise adherence, encouraging independent performance of ADLs, and implementing early mobilisation programmes to facilitate patient activity as soon as medically appropriate [1, 9, 10]. Despite increased evidence for the prevention of HAD, the prevalence of HAD has not decreased over the past three decades [11]. This underscores the need to develop supportive systems and environments that enable the effective implementation of HAD prevention in older adult patients.

In clinical settings, many of the above-mentioned hospital-associated factors for HAD are included in fundamental nursing care activities. However, time constraints and staffing shortages often result in missed or deprioritised fundamental care [10, 12]. Evidence suggests that lower nurse staffing levels in hospitals lead to adverse patient outcomes and increased mortality. Although an umbrella review investigating nurse staffing and patient outcomes showed insufficient evidence linking nurse staffing and HAD, the condition is considered a nursing-sensitive indicator [13]. Few studies have investigated the association between nurse staffing and HAD. One longitudinal survey of 1464 patients in the Italian medical unit focused on the skill mix of nursing staff, showing that a lower percentage of registered nurses (RNs) was associated with a higher risk of HAD [14]. Another cross-sectional survey of 873 older adults in two Israeli medical centres found no association between nurse-to-patient ratio and the older patients’ nursing-related care needs for mobility, continence care and food intake, although exposure to nurses with advanced training was associated with better performance of mobility and food intake [15]. However, the specific association between nurse staffing levels and HAD remains unclear. In addition, conditions during day and night shifts differ; staffing levels are typically lower during the night [16], nursing care activities vary across shifts [17], and patients often experience worse outcomes at night than those during the day [18, 19]. Despite this, most previous studies have used daily staffing measures, failing to distinguish between day and night shifts. This gap highlights the importance of analysing distinct staffing levels across shifts to better understand their impact on HAD.

Therefore, we examined the association between nurse staffing and HAD in older adults, using data divided into day and night shifts relative to typical staffing levels.

## Methods

This retrospective cohort study received ethical approval from the Medical Research Ethics Committee of Institute of Science Tokyo (approval no. M2023-113-02). The study was conducted in accordance with the Declaration of Helsinki, and the need for informed consent was waived owing to the anonymous nature of the data. Reporting followed the guidelines for the Reporting of Observational Studies in Epidemiology (STROBE).

### Data sources

We obtained patient discharge data and daily ward-level nursing administration data from 1 April 2019 to 31 March 2020, from nine acute care hospitals belonging to the National Hospital Organisation in Japan. We merged the two databases using dates, ward identifiers and hospital IDs. Electronic patient discharge data were obtained from the Diagnosis Procedure Combination (DPC) per diem payment system, a Japanese lump-sum payment system for acute care hospitals, introduced nationwide in 2003. The DPC data included patient clinical information such as patient demographics (e.g. date of birth, age), dates of admission and discharge, route of hospital admission, outcome at discharge, disease name, surgical procedure and daily scores for each evaluation item on the evaluation sheet for the severity of a patient’s condition and the need for medical/nursing care. Further details are provided elsewhere [20, 21].

Daily electronic ward-level nursing rostering data included the number of full-time-equivalent nurses on day and night shifts, the number of inpatients, the percentage of inpatients with more severe conditions, and the extent of a patient’s medical and nursing care needs. Hospitals are required to record the daily nurse staffing levels and the percentage of patients meeting these care needs in each ward and submit documents (named “Form No. 9”) annually to the Regional Bureau of Health and Welfare [22].

### Participants

We included 57 498 eligible admissions of patients aged 65 years or older across 84 general surgical or medical care wards in nine hospitals. Exclusion criteria included death case within 24 h of admission and missing data on any adjustment variables or nurse staffing levels at the ward level (Supplementary Appendix 1).

### Outcome

To assess functional status dependency changes from admission to discharge, we used the patient functional score from the Severity of a Patient’s Condition and Extent of a Patient’s Need for Medical/Nursing Care in Japan’s fee-schedule scheme [21]. Hospitals are required to exceed minimum staffing and care standards to qualify for this fee. Nurses trained in scoring evaluate the score daily at midnight (0:00) for every inpatient in the acute general care wards. Hospitals must ensure scoring accuracy, maintain patient progress records for at least two years, and undergo inspection by the Ministry of Health, Labour and Welfare.

The Severity of a Patient’s Condition and Extent of a Patient’s Need for Medical/Nursing Care were divided into three domains: (A) monitoring and treatment, (B) functional status and (C) treatment. We used domain (B) functional status, which consisted of seven items: turnover, transfer, oral care, feeding, personal dressing, ability to receive directions and engagement in dangerous behaviour. The total point ranges from 0 to 12 points (Supplementary Appendix 2). A 0-point scale indicated full independence; the higher the score, the greater the dependency. Most previous studies measured HAD using the Katz index or Barthel index [2, 3]. Compared with these existing scales, the patient’s functional status did not include bathing, toileting or continence but included cognitive function (Supplementary Appendix 2).

To measure the deterioration of the functional score during hospitalisation, we recorded the deterioration if the total score from admission to discharge was >0. If the total score remained the same or decreased, no deterioration was recorded.

### Nurse staffing

In this study, ‘nurse’ included RNs and licenced practical nurses. However, as of 2020, RNs accounted for over 98% of the total nursing staff across the nine hospitals, indicating that almost all were RNs according to the hospital’s report. We calculated (A) the mean patient-to-nurse ratios at the ward level during an individual’s hospitalisation for the entire day, as well as separately for day and night shifts, and (B) the annual mean patient-to-nurse ratios for each ward, both for the whole day and separately for the day and night shifts. To evaluate nurse staffing shortages relative to typical levels, we calculated the staffing shortfall as follows: (A) − (B), using previously described methodologies [23, 24]. A score greater than 0 indicates lower-than-typical staffing levels, while a score below 0 indicates better-than-typical staffing. Deviation from unit norms (mean or median) was used as a more comparable measure. In the absence of an objective measure of staffing requirement, this approach standardises the staffing measure and is preferable to an absolute measure of the patient-to-nurse ratio because different wards have different baseline staffing requirements. We calculated the deviation for the whole day and separately for day shifts (8 h: 9 a.m. to 5 p.m.) and night shifts (16 h: 5 p.m. to 9 a.m.).

### Adjustment variables

We selected the following covariates based on previous studies [7, 8]. The patient characteristics included age (up to quadratic term), sex, place of residence before admission (home, hospital or clinic, long-term care facility and others), Charlson Comorbidity Index (CCI) [25], surgery, weekend admission (or weekday admission), intensive care unit (ICU) experience within this admission (yes or no), dementia at admission (without dementia, mild or severe) using the score of ADL for older adults with dementia in the DPC data [26] and functional scores at admission. As for ward-level characteristics, we used the average functional status score of the inpatients and the percentage of inpatients with severe conditions during the patient’s hospitalisation.

### Statistical analysis

First, we described the characteristics of patients, outcomes, wards and hospitals, comparing them across the quartiles of the deviation in patient-to-nurse ratios relative to typical levels.

Second, we described the median and interquartile range (IQR) of the actual and annual mean of the patient-to-nurse ratio for the whole day, day shift or night shift.

Third, we examined the association between nurse staffing deviations and the occurrence of HAD using a three-level (individual, ward-level and hospital-level) multilevel logistic model with ward-level and hospital-level random intercepts that adjusted for the adjustment variables. The multilevel model was used to account for the correlations within the same ward and the same hospital. We separately performed analyses for each of the different staffing measures: a whole day (model 1), day shift (model 2) and night shift (model 3). For each model, the exposure variables determined based on the other two definitions were not included to avoid multicollinearity problems.

Fourth, we conducted the multilevel analysis (models 1–3) as a sensitivity analysis using the outcome of dependency at discharge (score ≥3).

Fifth, we assessed the impact of deviations in nurse staffing (patient-to-nurse ratio) relative to typical levels on patient outcomes, stratified by the primary medical condition for which patients were admitted. This analysis was conducted using Models 1–3 across the ten most common major diagnostic categories (MDCs) [20] (Supplementary Appendix 3).

Finally, we evaluated whether the differences in patient outcomes due to nurse staffing deviations (patient-to-nurse ratio) relative to typical levels were influenced by the illness severity across Models 1–3. Illness severity was defined by predicted in-hospital mortality based on patient characteristics, categorising into tertiles using logistic regression models [27, 28].

All reported p-values were two-sided, and statistical significance was set at *P* < .05. All analyses were performed using Stata MP ver.18 (StataCorp LLC., College Station, TX, USA).

## Results

### Patient characteristics and deterioration of functional dependency

We analysed 57 498 eligible admissions from 84 wards in nine hospitals. The median age of participants was 76 years (IQR 71–76), 23 981 (41.7%) were female and 30 507 (70%) were functionally independent at admission (Table 1 and Supplementary Appendix 4). In descending order, the most common MDCs were diseases of the digestive system, hepatobiliary system and pancreas (23.6%); circulatory system (16.8%); respiratory system (11.6%); kidney, urinary tract and male reproductive system (10.2%) and eye (7.0%) (Supplementary Appendix 3). Among these patients, functional dependency deterioration occurred in 15 458 hospital admissions (26.9%; Supplementary Appendix 4).

**Table 1:** Participants’ characteristics by overall and whole day nurse staffing deviation from typical levels.

			By deviation of patient-to-nurse ratio relative to typical							
			Better staffing than typical				Lower staffing than typical			
	Overall (*n* = 57 498)		First quartile (*n* = 14 375)		Second quartile (*n* = 14 375)		Third quartile (*n* = 14 375)		Fourth quartile (*n* = 14 375)	
Outcome										
Change in the functional status of dependency from admission to discharge (*n*, %)										
Unchanged or improved	42 040	73.1	10 613	73.83	10 446	72.67	10 367	72.12	10 614	73.84
Deterioration	15 458	26.9	3762	26.17	3928	27.33	4008	27.88	3760	26.16
Individual characteristics										
Age (median, IQR)	76	71–76	76	71–76	76	71–76	76	71–76	76	71–76
Sex (*n*, %)										
Male	33 517	58.3	8323	57.9	8500	59.1	8487	59.0	8207	57.1
Female	23 981	41.7	6052	42.1	5874	40.9	5888	41.0	6167	42.9
Surgery (*n*, %)	27 479	47.8	6770	47.1	6790	47.2	7056	49.1	6863	47.8
Length of hospital stay (day) (median, IQR)	9	4–9	6	3–6	10	4–10	11	5–11	10	5–10
ICU stay (*n*, %)	825	2.3	215	1.5	416	2.9	457	3.2	385	2.7
Weekend admission (n, %)	6540	11.4	1111	7.7	1628	11.3	1757	12.2	2044	14.2
CCI (*n*, %)										
0	23 870	41.5	6646	46.2	5881	40.9	5519	38.4	5824	40.5
1	4275	7.4	1056	7.4	1054	7.3	1111	7.7	1054	7.3
2	19 000	33.0	4332	30.1	4835	33.6	4960	34.5	4873	33.9
3 or over	10 353	18.0	2341	16.3	2604	18.1	2785	19.4	2623	18.3
Place of residence before admission (*n*, %)										
Home	54 993	95.6	13 931	96.9	13 714	95.4	13 638	94.9	13 710	95.4
Hospital or clinic	1436	2.5	248	1.7	372	2.6	450	3.1	366	2.6
Long-term care	1057	1.8	196	1.4	285	2.0	284	2.0	292	2.0
Others	12	0.0	0	0.0	3	0.0	3	0.0	6	0.0
Dementia at admission (*n*, %)										
Without dementia	51 719	90.0	13 095	91.1	12 929	90.0	12 831	89.3	12 864	89.5
Mild	3393	5.9	800	5.6	789	5.5	920	6.4	884	6.2
Severe	2386	4.2	480	3.3	656	4.6	624	4.3	626	4.4
Score of the functional status at admission (0–12) (mean, SD)	1.7	2.5	1.5	2.3	1.8	2.5	1.8	2.6	1.8	2.5
Ward-level characteristics										
Average score of the functional status (mean, SD)	3.7	1.1	3.8	1.1	3.7	1.1	3.7	1.1	3.6	1.2
Percentage of the severe inpatients (mean, SD)	34.3	13.6	35.8	14.1	34.4	13.1	33.9	13.5	33.0	13.4

Abbreviations: CCI, Charlson comorbidity index; ICU, intensive care unit; IQR, interquartile range; SD, standard deviation

The functional status was defined using the score of domain B (range 0–12 points) in the Severity of a Patient’s Condition and Extent of a Patient’s Need for Medical/Nursing Care in Japan.

### Nurse staffing

The median (IQR) of patient-to-nurse ratios in a day, day shift and night shift were 6.11 (5.54–6.59), 3.70 (3.34–3.90) and 9.38 (8.57–10.37), respectively. The median (IQR) deviation relative to the typical unit level was −0.21 (−0.64 to 0.20) for the patient-to-nurse ratio (Table 2). Patients exposed to the highest staffing levels (first quartile of deviation) had shorter lengths of stay (LOS), were less likely to be admitted on weekends, and were more likely to have a lower CCI score (Table 1).

**Table 2:** Actual and annual average of patient-to-nurse ratio in a day, day shift or night shift.

	Median	p25	p75
**Typical nurse staffing (average patient-to-nurse ratio) on the ward (*n* = 83)**			
A whole day	6.11	5.54	6.59
Day shift	3.70	3.34	3.90
Night shift	9.38	8.57	10.37
**Actual nurse staffing (patient-to-nurse ratio) (*n* = 57 498)**			
A whole day	5.84	5.18	6.45
Day shift	3.36	2.91	3.81
Night shift	9.42	8.43	10.36
**Deviation of patient-to-nurse ratio between the actual staffing and typical nurse staffing (*n* = 57 498)**			
A whole day	−0.21	−0.64	0.20
Day shift	−0.23	−0.56	0.09
Night shift	0.01	−0.51	0.49

### Nurse staffing and deterioration of functional dependency

When considering staffing across the whole day, multilevel logistic analysis showed that a higher patient-to-nurse ratio relative to the typical unit level was significantly associated with a higher risk of functional status deterioration (adjusted odds ratio [aOR] 1.068, 95% CI: 1.037–1.100, *P* < .001) (Table 3 and Supplementary Appendix 5). This relationship was also observed for each shift: day (aOR: 1.065, 95% CI: 1.024–1.108, *P* = .002) and night (aOR: 1.024, 95% CI: 1.006–1.042, *P* = .009) (Table 3 and Supplementary Appendix 5). This was also seen in the sensitivity analysis, where the outcome was dependency (score ≥3) (Supplementary Appendix 6).

**Table 3:** Nurse staffing deviation of patient-to-nurse ratio relative to typical and deterioration of dependency during hospitalisation (*n* = 57 498).

	OR	95% CI		*P*-value
Model 1: Whole day	1.068	1.037	1.100	<.001
Model 2: Day shift	1.065	1.024	1.108	.002
Model 2: Night shift	1.024	1.006	1.042	.009

Models 1–3: Multilevel logistic regression analysis adjusted for age, sex, place of resident before admission, surgery, CCI, dementia at admission, functional status at admission, weekend admission, ICU stay, average ADL score on the ward, percentage of severe inpatients on the ward. Nurse staffing deviation of patient-to-nurse ratio was calculated by the mean of the actual patient-to-nurse ratio during hospitalisation—the annual mean of the patient-to-nurse ratio on the ward.

Abbreviations: OR, odds ratio; CI, confidence interval; CCI, Charlson Comorbidity Index; ICU, intensive care unit; ADL, activities of daily living.

Other variables associated with a higher risk of HAD included older age, dementia, residence in settings other than at home, higher CCI scores without surgery, weekend admission, ADL score at admission and average dependency in the ward.

### Nurse staffing and deterioration of functional dependency in terms of primary diagnoses and severity of illness

**Figure 1:**
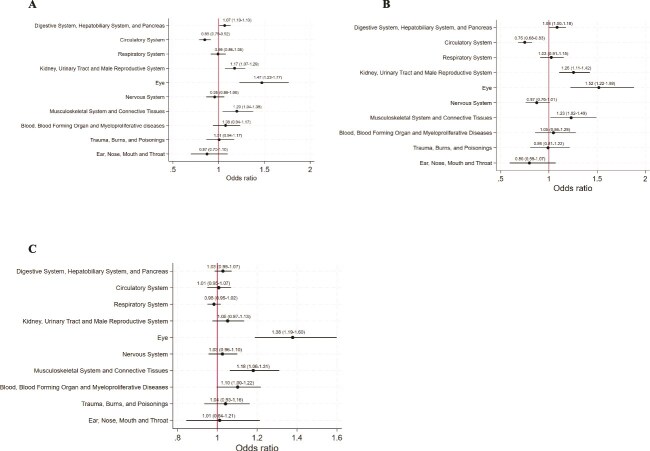
Analysis of the ten most common MDCs in a whole day (a), day shift (b) and night shift (c). Multilevel logistic regression analysis adjusted for age, sex, place of resident before admission, surgery, CCI, dementia at admission, functional status at admission, weekend admission, ICU stay, average ADL score on the ward, and percentage of severe inpatients on the ward. Nurse staffing deviation of the patient-to-nurse ratio was calculated by the mean of the actual patient-to-nurse ratio during hospitalisation—the annual mean of the patient-to-nurse ratio on the ward. Abbreviations: MDCs, major diagnostic categories; CCI, Charlson Comorbidity Index; ICU, intensive care unit; ADL, activities of daily living.

**Figure 2:**
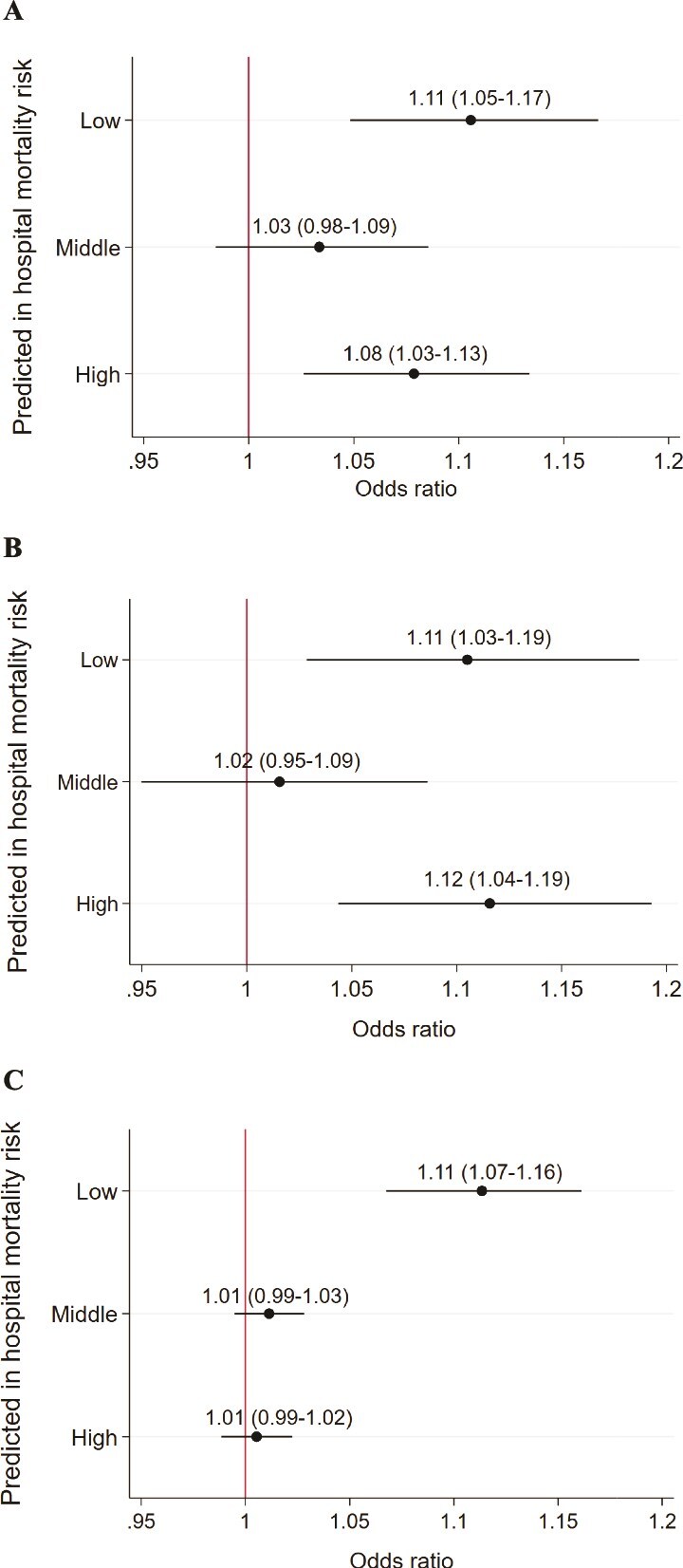
Analysis by predicted mortality risk in a whole day (a), day shift (b) and night shift (c). Multilevel logistic regression analysis adjusted for age, sex, place of resident before admission, surgery, CCI, dementia at admission, functional status at admission, weekend admission, ICU stay, average ADL score on the ward, and percentage of severe inpatients on the ward. Nurse staffing deviation of the patient-to-nurse ratio was calculated by the mean of the actual patient-to-nurse ratio during hospitalisation—the annual mean of the patient-to-nurse ratio on the ward. Abbreviations: CCI, Charlson Comorbidity Index; ICU, intensive care unit; ADL, activities of daily living.

Subgroup analysis by primary diagnosis across the ten common MDCs (Figure 1) showed consistent relationships between higher patient-to-nurse ratios and functional dependency deterioration across most MDCs except for circulatory diseases, both for the whole day and day shift. In analysis stratified by patient illness severity tertiles, a higher patient-to-nurse ratio relative to typical levels was associated with increased risk of HAD for the whole day, day shift and night shift in all tertile groups (Figure 2).

## Discussion

To our knowledge, this study is the first to show that older adults exposed to lower-than-typical nurse staffing levels during either the day or night shift were more likely to experience functional decline in acute hospital settings. Only one previous study has examined the relationship between nurse staffing levels and functional status in older adults, finding no statistically significant association between patient-to-nurse ratios (daily average) and outcomes such as mobility, continence care or food intake, using classification decision tree analysis [15]. However, the study only assessed these outcomes within 2 days of admission rather than tracking functional changes during hospitalisation. Our study contributes new insights, demonstrating that adequate staffing levels during both day and night shifts may help prevent HAD in older adults. This finding is based on a large sample of patient discharge and ward-level nursing roster data. Several potential explanations support these findings.

One potential explanation is that adequate nurse staffing may facilitate the implementation of essential nursing activities that are crucial for preventing HAD. For HAD prevention, nurses are expected to encourage mobility and environmental adjustments (e.g. providing easy access to a bedside chair and standing orders for patients to get out of bed and into a chair) and encourage patients to perform activities under supervision during fundamental care [1, 9]. However, in environments with frequent understaffing, nurses tended to miss these fundamental nursing care activities, such as mobilisation, activating or rehabilitating care or oral hygiene, and continence training [12]. In particular, there are typically fewer RNs on a night shift for an acute hospital ward than those on a day shift because the patients are asleep during night. However, considering the 16 h duration of the night shift, especially given that patients will not be asleep during its entirety, fundamental nursing care still tends to be deprioritised over life-threatening clinical care in the night shift [16]. Adequate staffing enables nurses to provide fundamental care activities to reduce the risk of functional decline and HAD in older adults [10]. While increasing the number of nurses is important, there may also be a need to add training for nurses to care for older adults to maintain their ADLs, as previous studies have reported that additional training for nurses is associated with patient mobility [15]. Similarly, the Acute Care for Elders model of care delivered by interdisciplinary teams, including advanced practice nurses, has effectively maintained ADLs and prevented functional decline [29].

Another potential explanation is that ensuring appropriate nursing staff may help reduce adverse events and shorten the LOS, ultimately contributing to the prevention of HAD in older adults. A meta-analysis has shown that increased RN staffing is associated with a lower risk of surgical wound infection (OR: 0.15, 95% CI: 0.03–0.82) and nosocomial bloodstream infection (OR: 0.64, 95% CI: 0.46–0.89) [30]. An umbrella review summarising 201 primary studies found moderate evidence linking lower nurse staffing levels to adverse events such as medication errors, pneumonia and respiratory failure, all of which can prolong hospital stays [13]. Additionally, a 1-day increase in LOS was associated with an 8% increase in the risk of HAD [8]. This evidence highlights the complex pathways through which adequate ward-level staffing may help prevent HAD in older adults by reducing adverse events and shortening LOS.

### Limitations

This study has some limitations. First, we used an original score system to measure HAD due to data availability. However, the accuracy of these scores is ensured by their evaluation by trained nurses who undergo audits for reimbursement under Japan’s National Health Insurance. In our study, 26.9% of older adults experienced HAD, with a similar prevalence reported in previous studies using the Katz index or BI [2, 3]. Second, although we controlled for a range of confounding factors and normalised staffing levels to ward averages, unmeasured confounders may still bias our results, such as the presence of senior staff members with expertise in gerontology, existence of a multidisciplinary team, or organisational culture [9]. However, we accounted for ward- or hospital-level characteristics as fixed effects in the analysis. Third, we used the mean nurse staffing level during hospitalisation to calculate exposure, and did not take variations in daily nurse staffing levels into account in the analysis. Fourth, there is no standardised time block definition for the night shifts across different countries. In the USA, the night shifts typically run between 7 p.m. and 7 a.m. [31], spanning 8–12 h. In Japan, night shifts last 16 h, overlapping partially with day shifts as defined in other countries. Finally, the data used in this study were collected during the pre-COVID period in Japan. Further studies conducted during the post-COVID period are necessary to examine the relationship between nurse staffing and HAD in the current healthcare environment. However, as the basic nurse staffing standards in the fee schedule have remained unchanged since the pre-COVID period, the findings of this study could be generalised to the current post-COVID situation.

Regardless of these limitations, our study highlights that adequate nurse staffing allocated to both daytime and night-time shifts serves as a modifiable factor for reducing HAD in older adults.

In conclusion, insufficient nurse staffing during the whole day, day shift or night shift was associated with a higher risk of functional dependency deterioration in older adults admitted to acute care hospitals. Attention to the deviations from the usual staffing levels for both day and night shifts may be important in efforts to reduce the risk of HAD in older adults.

## Supplementary Material

aa-25-0426-File004_afaf217

aa-25-0426-File005_afaf217

aa-25-0426-File006_afaf217

aa-25-0426-File007_afaf217

aa-25-0426-File008_afaf217

aa-25-0426-File009_afaf217

## Data Availability

All datasets in this study have ethical or legal restrictions for public deposition due to inclusion of sensitive information from the human subjects.
