# Supplementary figures and images for: Day and night nurse staffing levels and hospital-associated disability in older adults in Japan: a retrospective cohort study
Source: Age Ageing. 2025 Aug 6;54(8):afaf217. doi: 10.1093/ageing/afaf217 (PMC12341895; doi:10.1093/ageing/afaf217)

## Appendix 1. Flowchart of the sample selection

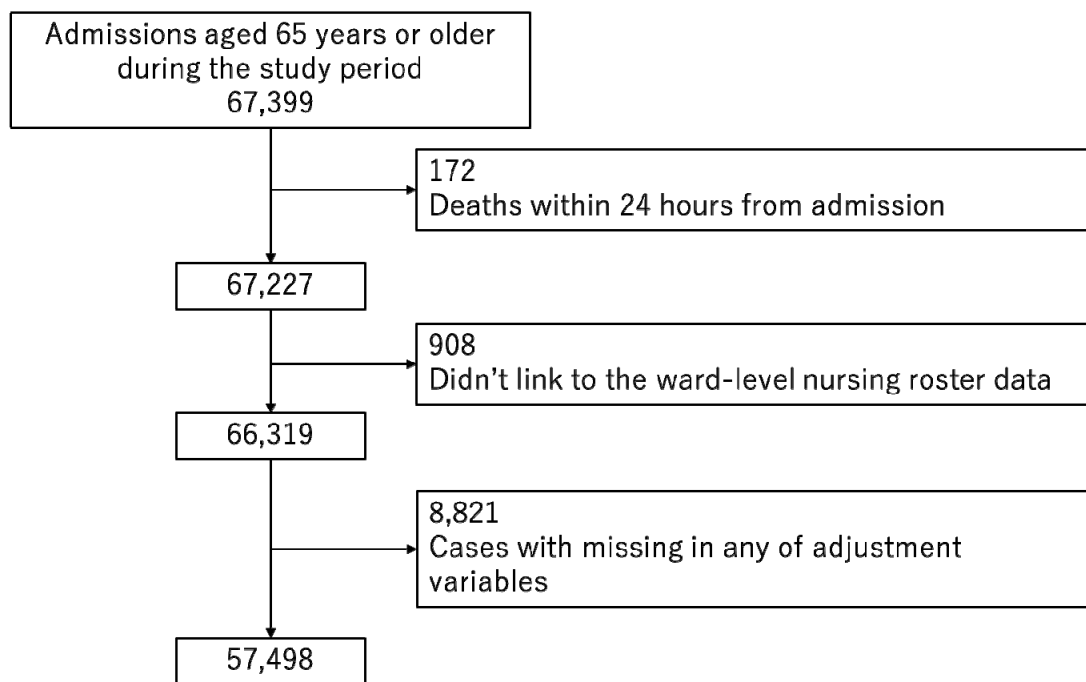

Supplement: aa-25-0426-File004_afaf217 [file aa-25-0426-file004_afaf217.pdf]
