# Supplementary material for: Day and night nurse staffing levels and hospital-associated disability in older adults in Japan: a retrospective cohort study
Source: Age Ageing. 2025 Aug 6;54(8):afaf217. doi: 10.1093/ageing/afaf217 (PMC12341895; doi:10.1093/ageing/afaf217)
Supplement: aa-25-0426-File005_afaf217 [file aa-25-0426-file005_afaf217.pdf]

Appendix 2. Comparison among scales for activities of daily living (ADLs).

Items		Katz Index[1]<br>(6 = High (patient independent) 0 = Low (patient very dependent))	Barthel Index [2](0-100)	Severity of a Patient's condition and extent of a Patient's need of medical/nursing care[3]
ADL	BATHING	0 = Need help with bathing more than one part of the body, getting in or out of the tub or shower. Requires total bathing<br>1 = Bathes self completely or needs help in bathing only a single part of the body such as the back, genital area or disabled extremity.	0 = dependent<br>5 = independent (or in shower)	NA
	DRESSING	0 =Needs help with dressing self or needs to be completely dressed.<br>1 = Get clothes from closets and drawers and puts on clothes and outer garments complete with fasteners. May have help tying shoes.	0 = dependent<br>5 = needs help but can do about half unaided<br>10 = independent (including buttons, zips, laces, etc.)	0 = Without assistance<br>1 =Partly assisted<br>2 =Fully assisted
	GROOMING	NA	0 = needs to help with personal care<br>5 = independent face/hair/teeth/shaving (implements provided)	<Oral care><br>0 = Without assistance<br>1 = With assistance
	TOILETING	0 =Needs help with dressing self or needs to be completely dressed.<br>1 = Goes to toilet, gets on and off, arranges clothes, cleans genital area without help	0 = dependent<br>5 = needs some help, but can do something alone<br>10 = independent (on and off, dressing, wiping)	NA
	CONTINENCE	0 =Is partially or totally incontinent of bowel or bladder<br>1 = Exercises complete self control over urination and defecation.	<Bowel control><br>0 = incontinent (or needs to be given enemas)<br>5 = occasional accident<br>10 = continent<br><Bladder control><br>0 = incontinent, or catheterized and unable to manage alone<br>5 = occasional accident<br>10 = continent	NA
	FEEDING	0 =Needs partial or total help with feeding or requires parenteral feeding.<br>1 = Gets food from plate into mouth without help. Preparation of food may be done by another person.	0 = unable<br>5 = needs help cutting, spreading butter, etc., or requires modified diet<br>10 = independent	0 = Without assistance<br>1 = Partly assisted<br>2 = Fully assisted
	TRANSFERRING	0 =Needs help in moving from bed to chair or requires a complete transfer<br>1 = Moves in and out of bed or chair unassisted. Mechanical transfer aids are acceptable	0 = unable, no sitting balance<br>5 = major help (one or two people, physical), can sit<br>10 = minor help (verbal or physical)<br>15 = independent	<Turn over><br>0 = Can do<br>1 = Can do if grabbing something<br>2 =Cannot<br><Transfer><br>0 = Without assistance<br>1 = Partly assisted<br>2 = Fully assisted
	Mobility on level surfaces	NA	0 = immobile or < 50 yards<br>5 = wheelchair independent, including corners, > 50 yards<br>10 = walks with help of one person (verbal or physical) > 50 yards<br>15 = independent (but may use any aid; for example, stick) > 50 yards	NA
	STAIRS	NA	0 = unable<br>5 = needs help (verbal, physical, carrying aid)<br>10 = independent	NA
Cognitive function	Able to receive directions on medical care and treatment	NA	NA	0 = Yes<br>1 = No
	Engaged in dangerous behavior	NA	NA	0 = No<br>1 = Yes

1. Katz S, Ford AB, Moskowitz RW, Jackson BA, Jaffe MW. STUDIES OF ILLNESS IN THE AGED. THE INDEX OF ADL: A STANDARDIZED MEASURE OF BIOLOGICAL AND PSYCHOSOCIAL FUNCTION. JAMA. 1963;185:914–919. doi: 10.1001/jama.1963.03060120024016.
2. Mahoney FI, Barthel DW. FUNCTIONAL EVALUATION: THE BARTHEL INDEX. Md State Med J. 1965;14:61–65.
3. Hayashida K, Moriwaki M, Murakami G. Evaluation of the condition of inpatients in acute care hospitals in Japan: A retrospective multicenter descriptive study. Nurs Health Sci. 2022;24(4):811–819. doi: 10.1111/nhs.12980.
