# Supplementary material for: Day and night nurse staffing levels and hospital-associated disability in older adults in Japan: a retrospective cohort study
Source: Age Ageing. 2025 Aug 6;54(8):afaf217. doi: 10.1093/ageing/afaf217 (PMC12341895; doi:10.1093/ageing/afaf217)
Supplement: aa-25-0426-File006_afaf217 [file aa-25-0426-file006_afaf217.pdf]

Appendix 3. Characteristics of patient's primay diagnosis by the major diagnostic categories (MDCs)

n=57,498

Major diagnostic categories (MDCs) codes	Disease name	n	%
1	Diseases and Disorders of the Nervous System	3,797	6.6
2	Diseases and Disorders of the Eye	4,006	7.0
3	Diseases and Disorders of the Ear, Nose, Mouth and Throat	1,474	2.6
4	Diseases and Disorders of the Respiratory System	6,651	11.6
5	Diseases and Disorders of the Circulatory System	9,683	16.8
6	Diseases and Disorders of the Digestive System, Hepatobiliary System, and Pancreas	13,595	23.6
7	Diseases and Disorders of the Musculoskeletal System and Connective Tissues	3,376	5.9
8	Diseases and Disorders of the Skin and Subcutaneous Tissue	952	1.7
9	Diseases and Disorders of the Breast	507	0.9
10	Diseases and Disorders of the Endocrine, Nutritional and Metabolic System	1,358	2.4
11	Diseases and Disorders of the Kidney, Urinary Tract and Male Reproductive System	5,863	10.2
12	Diseases and Disorders Pertaining to the Female Reproductive System, Pregnancy, Childbirth, and Puerperium	535	0.9
13	Diseases and Disorders of the Blood, Blood Forming Organ and Myeloproliferative Diseases and Disorders	2,545	4.4
14	Neonatal Diseases and Disorders	0	0.0
15	Pediatric Diseases and Disorder	33	0.1
16	Trauma, Burns, and Poisonings	2,246	3.9
17	Mental Diseases and Disorders	25	0.0
18	Other Diseases and Disorders	852	1.5
