# Supplementary material for: Day and night nurse staffing levels and hospital-associated disability in older adults in Japan: a retrospective cohort study
Source: Age Ageing. 2025 Aug 6;54(8):afaf217. doi: 10.1093/ageing/afaf217 (PMC12341895; doi:10.1093/ageing/afaf217)
Supplement: aa-25-0426-File007_afaf217 [file aa-25-0426-file007_afaf217.pdf]

Appendix 4. Change in the functional score from admission to discharge

		Functional score at discharge									Total
		0	1	2	3	4	5	6	7	8 or over	
Functional score at admission	0	21,120	3829	1,554	1,357	739	675	396	337	500	30,507
		69.2	12.6	5.1	4.5	2.4	2.2	1.3	1.1	1.6	100
	1	2,125	2983	604	408	199	173	101	73	67	6,733
		31.6	44.3	9.0	6.1	3.0	2.6	1.5	1.1	1.0	100
	2	1,167	616	1,107	412	211	164	109	62	52	3,900
		29.9	15.8	28.4	10.6	5.4	4.2	2.8	1.6	1.3	100
	3	1,001	521	383	1,202	329	271	145	83	76	4,011
		25.0	13.0	9.6	30.0	8.2	6.8	3.6	2.1	1.9	100
	4	726	367	295	358	722	369	239	160	97	3,333
		21.8	11.0	8.9	10.7	21.7	11.1	7.2	4.8	2.9	100
	5	480	277	228	306	336	885	305	189	203	3,209
		15.0	8.6	7.1	9.5	10.5	27.6	9.5	5.9	6.3	100
	6	249	118	118	130	144	272	436	205	213	1,885
		13.2	6.3	6.3	6.9	7.6	14.4	23.1	10.9	11.3	100
	7	155	81	56	77	90	231	196	339	251	1,476
		10.5	5.5	3.8	5.2	6.1	15.7	13.3	23.0	17.0	100
	8 or over	129	56	54	68	83	238	297	359	1160	2,444
		5.3	2.3	2.2	2.8	3.4	9.7	12.2	14.7	47.5	100.0
Total		27,152	8848	4,399	4,318	2,853	3,278	2,224	1,807	2,619	57,498
		47.2	15.4	7.7	7.5	5.0	5.7	3.9	3.1	4.6	100
