# Supplementary material for: Day and night nurse staffing levels and hospital-associated disability in older adults in Japan: a retrospective cohort study
Source: Age Ageing. 2025 Aug 6;54(8):afaf217. doi: 10.1093/ageing/afaf217 (PMC12341895; doi:10.1093/ageing/afaf217)
Supplement: aa-25-0426-File008_afaf217 [file aa-25-0426-file008_afaf217.pdf]

Appendix 5. Results of multilevel analysis for the deterioration in the total functional status dependency

	Model 1				Model 2				Model 3			
	OR	95% CI		P-value	OR	95% CI		P-value	OR	95% CI		P-value
Deviation between the actual patient-to-nurse ratio and the annual mean on the ward*												
A whole day	1.068	1.037	1.100	<0.001								
Day-shift					1.065	1.024	1.108	0.002				
Night-shift									1.024	1.006	1.042	0.009
**Individual variables**												
Sequared age	1.001	1.001	1.001	<0.001	1.001	1.001	1.001	<0.001	1.001	1.001	1.001	<0.001
Age	0.891	0.846	0.938	<0.001	0.891	0.846	0.938	<0.001	0.890	0.845	0.938	<0.001
Sex (ref. male)												
Female	1.022	0.980	1.064	0.310	1.021	0.980	1.064	0.312	1.022	0.981	1.065	0.293
Dementia (ref. without dementia)												
Mild	1.989	1.832	2.160	<0.001	1.988	1.830	2.159	<0.001	1.989	1.832	2.160	<0.001
Severe	3.095	2.802	3.419	<0.001	3.093	2.800	3.416	<0.001	3.098	2.805	3.422	<0.001
Place of residence before admission (ref. home)												
Hospital or clinic	1.978	1.744	2.242	<0.001	1.977	1.744	2.242	<0.001	1.984	1.750	2.250	<0.001
Long-term care	1.820	1.570	2.111	<0.001	1.820	1.569	2.111	<0.001	1.819	1.568	2.109	<0.001
Others	1.005	0.208	4.850	0.995	1.012	0.210	4.876	0.988	1.025	0.213	4.940	0.976
CCI												
1	1.140	1.054	1.234	0.001	1.141	1.054	1.234	0.001	1.143	1.056	1.237	0.001
2	1.057	1.007	1.110	0.026	1.058	1.007	1.111	0.024	1.059	1.009	1.113	0.021
3 or over	1.219	1.149	1.293	<0.001	1.220	1.150	1.294	<0.001	1.222	1.152	1.297	<0.001
Surgery	0.746	0.714	0.780	<0.001	0.747	0.715	0.780	<0.001	0.748	0.716	0.782	<0.001
Weekend admission	1.090	1.025	1.160	0.006	1.089	1.023	1.159	0.008	1.101	1.035	1.172	0.002
ICU stay	1.170	1.024	1.336	0.021	1.170	1.024	1.336	0.021	1.179	1.032	1.346	0.015
Score of the functional status at ad	0.780	0.771	0.788	<0.001	0.780	0.771	0.788	<0.001	0.781	0.772	0.789	<0.001
**Ward-level variables**												
Average score of the functional status	1.232	1.178	1.287	<0.001	1.230	1.177	1.286	<0.001	1.229	1.176	1.285	<0.001
Percentage of the severe inpatients	0.997	0.995	1.000	0.071	0.997	0.994	1.000	0.055	0.997	0.994	1.000	0.051
_cons	5.547142	0.7336029	41.94474	0.097	5.597642	0.7403534	42.32248	0.095	5.709134	0.7549789	43.17235	0.091
**Random effect part**												
Ward level (Variance)	6.35E-35				2.00E-29				2.35E-32			
Hospital level (Variance)	0.3222301				0.3249248				0.329867			

CCI: charlson comorbidity index, ICU: Intensive care unit
*Nurse staffing deviation of patient-to-nurse ratio was calculated by the mean of the actual patient-to-nurse ratio during hospitalization - the annual mean of the patient-to-nurse ratio on the ward.
