# Supplementary material for: Day and night nurse staffing levels and hospital-associated disability in older adults in Japan: a retrospective cohort study
Source: Age Ageing. 2025 Aug 6;54(8):afaf217. doi: 10.1093/ageing/afaf217 (PMC12341895; doi:10.1093/ageing/afaf217)
Supplement: aa-25-0426-File009_afaf217 [file aa-25-0426-file009_afaf217.pdf]

Appendix 6. Results of multilevel analysis for the dependency at discharge (functional score ≥3) .

n=57,498

	Model 1				Model 2				Model 3			
	OR	95% CI		P-value	OR	95% CI		P-value	OR	95% CI		P-value
**Deviation between the actual patient-to-nurse ratio and the annual mean on the ward***												
A whole day	1.06	1.03	1.09	<0.001								
Day-shift					1.09	1.05	1.14	<0.001				
Night-shift									1.01	1.00	1.02	0.185
**Individual variables**												
Sequared age	1.00	1.00	1.00	<0.001	1.00	1.00	1.00	<0.001	1.00	1.00	1.00	<0.001
Age	0.71	0.67	0.76	<0.001	0.71	0.67	0.76	<0.001	0.71	0.67	0.75	<0.001
Sex (ref. male)												
Female	0.92	0.89	0.97	<0.001	0.93	0.89	0.97	<0.001	0.92	0.89	0.97	<0.001
Dementia (ref. without dementia)												
Mild	2.91	2.68	3.16	<0.001	2.90	2.67	3.15	<0.001	2.91	2.68	3.16	<0.001
Severe	6.45	5.77	7.21	<0.001	6.44	5.76	7.20	<0.001	6.46	5.78	7.22	<0.001
Place of residence before admission (ref. home)												
Hospital or clinic	4.98	4.39	5.65	<0.001	4.97	4.38	5.63	<0.001	5.00	4.41	5.67	<0.001
Long-term care	5.51	4.60	6.60	<0.001	5.50	4.59	6.58	<0.001	5.52	4.61	6.61	0.000
Others	3.04	0.83	11.17	0.094	3.02	0.82	11.08	0.096	3.11	0.85	11.43	0.087
CCI (ref. 0)												
1	1.20	1.10	1.30	<0.001	1.20	1.10	1.30	<0.001	1.20	1.10	1.30	<0.001
2	0.98	0.93	1.03	0.387	0.98	0.93	1.03	0.374	0.98	0.93	1.03	0.433
3 or over	1.26	1.18	1.34	<0.001	1.26	1.18	1.34	<0.001	1.26	1.19	1.34	<0.001
Surgery	0.52	0.50	0.55	<0.001	0.52	0.50	0.55	<0.001	0.52	0.50	0.55	<0.001
Weekend admission	1.28	1.21	1.37	<0.001	1.28	1.20	1.36	<0.001	1.30	1.22	1.38	<0.001
ICU stay	1.57	1.37	1.79	<0.001	1.56	1.37	1.78	<0.001	1.58	1.38	1.80	<0.001
**Ward-level variables**												
Average score of the functional status	1.59	1.51	1.66	<0.001	1.58	1.51	1.66	<0.001	1.58	1.51	1.66	<0.001
Percentage of the severe inpatients	1.00	0.99	1.00	0.048	1.00	0.99	1.00	0.047	1.00	0.99	1.00	0.027
_cons	3505.82	376.17	32673.21	<0.001	3478.90	373.25	32425.15	<0.001	3610.72	387.62	33633.79	<0.001
**Random effect part**												
Ward level (Variance)	0.33	0.21	0.52		0.33	0.21	0.52		0.34	0.22	0.52	
Hospital level (Variance)	0.05	0.01	0.36		0.05	0.01	0.35		0.03	0.00	0.30	

OR, odds ratio; CCI, Charlson comorbidity score, ICU, intensive care unit

*Nurse staffing deviation of patient-to-nurse ratio was calculated by the mean of the actual patient-to-nurse ratio during hospitalization - the annual mean of the patient-to-nurse ratio on the ward.
